# The prognostic value of Foxp3+ tumor-infiltrating lymphocytes in patients with glioblastoma

**DOI:** 10.1007/s11060-013-1314-0

**Published:** 2013-11-26

**Authors:** Qi Yue, Xin Zhang, Hong-xing Ye, Yin Wang, Zun-guo Du, Yu Yao, Ying Mao

**Affiliations:** 1 Department of Neurosurgery, Huashan Hospital, Fudan University, No. 12 Mid Wulumuqi Road, Shanghai, 200040 People’s Republic of China; 2 Department of Neuropathology, Huashan Hospital, Fudan University, Shanghai, People’s Republic of China; 3 Department of Pathology, Huashan Hospital, Fudan University, Shanghai, People’s Republic of China

**Keywords:** Foxp3, Tumor-infiltrating lymphocyte, Regulatory T cell, Glioblastoma, Prognosis

## Abstract

Forkhead box protein 3 (Foxp3) is known as a specific marker for regulatory T cells which contribute to immunosuppression in tumor microenvironment. However, existing studies regarding clinical significance of Foxp3+ tumor-infiltrating lymphocytes (TILs) in glioblastoma (GBM) remained discrepant. In this study, we aimed to explore whether this subtype of TILs correlated with prognosis in patients with GBM. Foxp3+ TILs as well as CD8+ ones were detected by immunohistochemistry on paraffin-embedded tumor samples from 62 patients. Staining for p53, MGMT and Ki-67 were also performed. The correlation of TIL subtypes with clinicopathologic features were analyzed. Progression-free survival (PFS) and overall survival (OS) were estimated by Kaplan–Meier method and compared using log-rank test. Independent prognostic factors for PFS and OS were determined through univariate and multivariate analysis. Significant correlation was found between Foxp3 and CD8 expression (*P* = 0.003), but not between TIL subtypes and clinicopathologic characteristics. Patients with higher density of Foxp3+ TILs showed relatively shorter PFS (*P* < 0.001) and OS (*P* = 0.003) whereas patients with higher density of CD8+ TILs obtained no significant differences in survival. Survival analysis based on molecular classifications further clarified these predictive values. Univariate and multivariate analysis revealed that frequency of Foxp3+ TILs was probably associated with both PFS (*P* = 0.002) and OS (*P* = 0.003). In conclusion, the results suggest that Foxp3 positive infiltrates could provide an independent predictive factor in GBM.

**Electronic supplementary material:**

The online version of this article (doi:10.1007/s11060-013-1314-0) contains supplementary material, which is available to authorized users.

## Introduction

Glioma is one of the most prevalent tumors in central nervous system and accounts for more than 50 % of primary intracranial neoplasms in adults. Given that combination of surgery, radiotherapy and chemotherapy has made rapid advances in recent years, the median survival of glioblastoma (GBM, WHO grade IV glioma) remains at 14.6 months and 2-year survival rate is only 26.5 % [1]. It has been realized by more and more researchers that a final cure of this malignancy may depend on better understanding of the biological behavior [2]. For this, diverse theories are emerging to explain GBM’s malignant characteristics, among which the mechanism of immune escape seems extremely prospective and attracts widely attentions.

Since the brain was not regarded as an immunoprivileged organ, various subtypes and immunologic features of tumor infiltrating lymphocytes (TILs) have been investigated over the past decade [3]. Besides well-known CD8+ and CD4+ T cells, regulatory T cells (Tregs), initially recognized by CD4+ CD25+ profile, are also considered to play an important part in suppressing immune response and promoting tumor invasion [4, 5]. Many studies have revealed Tregs’ upregulation in peripheral blood of patients suffering from liver, gastric, breast and esophagus cancer, as well as infiltration in tumor microenvironment [6–8]. Forkhead box protein 3 (Foxp3), primarily identified as a transcription factor for Treg, is likely to be responsible for its immunosuppressive function and has popularly become its single marker in cancer research [9]. To evaluate Tregs’ prognostic value, research on the correlation between the density of Foxp3+ TILs in tumor tissue and clinical outcome was performed in nearly all kinds of tumors, leading to both positive and negative results [10]. As for GBM, the discrepancy also exists that two early studies denied Foxp3+ TILs’ association with prognosis whereas a recent study demonstrated patients with higher Foxp3 expression had shorter survival time than those with lower expression [11–13]. Hence, existing data are still limited and more obtained from larger number of cases are still in need for further elucidating whether Foxp3+ TILs can be used as a prognostic factor for GBM patients. In addition, the relationship between Foxp3 and other GBM molecular markers such as p53, MGMT, Ki-67 is also unclear.

Our early research has focused on the interaction between glioma stem-like cells (GSCs) and microglias/macrophages through negative costimulatory molecules such as B7 family, and revealed potential mechanism underlying GBM’s immune escape induced by GSCs [14, 15]. Based on these, we now push our study forward to the TIL-centered downstream of the mechanism, by exploring the immunosuppressive Foxp3+ TILs at first. Here we detected the expression of Foxp3 in tumor tissues obtained from 62 GBM patients and examined its predictive significance for clinical outcome.

## Materials and methods

### Patients and tissue samples

From 2006 to 2010, all newly diagnosed GBM patients who underwent tumor resection at Huashan Hospital by one neurosurgical team were enrolled in our study. Histopathological diagnosis was performed by experienced neuropathologists according to World Health Organization (WHO) guidelines [16]. All patients received radiotherapy (fractionated focal irradiation 2 Gy/day, 5 days/week for 6 weeks, 60 Gy totally) and temozolomide therapy (75 mg/m^2^ day, 7 days/week throughout radiotherapy, followed by 150–200 mg/m^2^ for 5 days during each cycle, 28 days per cycle for 6 cycles) postoperatively. Informed consent was obtained from all patients and the protocol for this study was approved by the Institutional Review Board of Huashan Hospital.

Detailed clinicopathologic data during hospitalization were collected from medical records for each patient, including sex, age, tumor location, tumor diameter and the extent of tumor resection. Follow up was completed at August, 2012, collecting information about following treatments, recurrence and survival status. Progression-free survival (PFS) was defined as the time interval from diagnosis to first tumor progression or recurrence. Overall survival (OS) was measured from diagnosis to death or censor.

### Immunohistochemical staining

Paraffin sections (4 μm) of GBM were mounted on silanized slides, dewaxed at 56 °C for 30 min, deparaffinized with xylenes and rehydrated through ethanol. Peroxidase activity was quenched with 3 % hydrogen peroxidase for 15 min and non-specific background staining was eliminated by blocking buffer (2 % goat serum, 0.2 % Triton X-100, 0.1 % bovine serum albumin in phosphate-buffered saline) at room temperature for 1 h. Primary antibodies against Foxp3 (1:50; Abcam), CD8 (prediluted; Dako), p53 (1:400; Abcam), MGMT (1:100; Abcam) and Ki-67 (1:50; Abcam) were then applied overnight at 4 °C. After a series of washing, immunodetection was performed using labeled polymer horseradish peroxidase mouse antibody (EnVision+ system, Dako). Slides were subsequently rinsed with deionized water, visualized with 3,3′-diaminobenzidine for 10 min and counterstained with Mayers hematoxylin. The negative controls were obtained by omitting primary antibodies. Human lymph nodes were used as a positive control for Foxp3, CD8, MGMT and Ki-67, while human gastric adenocarcinoma tissue was chosen for p53.

### Evaluation of immunohistochemical variables

Slides were reviewed under Nikon eclipse TE2000-S microscope by two independent observers (Y. W. and Q. Y.) blind to clinical outcomes and captured using Image-Pro Plus 5.0 software. For detection of CD8 expression, lymphocytes with clear membranous staining were regarded as positive ones. As to Foxp3 analysis, lymphocytes which showed evident nuclear staining were interpreted as immunoreactive cells. The numbers of CD8+ and Foxp3+ cells were counted under 5 randomly selected fields at high magnification (×400) by each observer. With an eyepiece grid covering 0.24 mm^2^, positively stained lymphocytes were finally expressed as cell numbers per mm^2^ of tumor tissue [17].

Labeling index, which meant the percentage of nuclear positive cells under high-powered field (HPF), was applied to evaluate expression patterns of p53 [18], MGMT [19] and Ki-67 [20]. The cutoff values were 5 % for p53, 10 % for MGMT and 20 % for Ki-67, respectively. Cases with labeling index above cutoff value were categorized under positive groups.

### Statistical analysis

All data analysis except Cox regression models was performed with GraphPad Prism 5.0 software. Clinicopathologic parameters were treated as discrete variables and the TIL distributions were compared using the Mann–Whitney *U*-test. Survival analysis was performed with the Kaplan–Meier method and significance was confirmed by the log-rank test. Cox proportional hazards regression models were constructed in both univariate and multivariate analysis by SPSS 20.0 software. Statistical significance was established at *P* < 0.05.

## Results

### Clinicopathologic data of the patients

Forty-three male and nineteen female patients were enrolled in this study, and the median age was 56 years old (range 13–77). The tumors were located in various regions and gross total resection was achieved in 50 patients. According to our categorization, more than one-third of the patients were positive for p53, MGMT or Ki-67. The median follow-up duration was 17.5 months (range 3–77) and no patient was lost. At the last follow-up, only four were still alive and two of them had experienced tumor recurrence.

### Correlation of TIL parameters with clinicopathologic characteristics

**Fig. 1:**
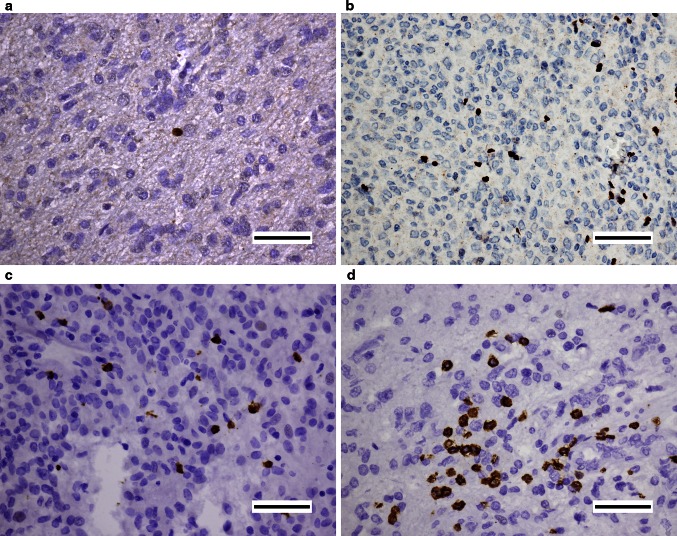
Representative micrographs of Foxp3 and CD8 expression in GBM. Low (**a**), high (**b**) numbers of Foxp3+ cells with nuclear stain and low (**c**), high (**d**) numbers of CD8+ cells with membrane stain were shown respectively. *Scale bars* 50 μm

Different intensities of Foxp3 and CD8 expression were shown in Fig. 1. The density of Foxp3+ TILs was statistically lower than that of CD8+ ones (8.6 vs 49.6 mm^2^, *P* < 0.001). As the two parameters were significantly correlated (Supplementary Fig. 1), we set the Foxp3+/CD8+ ratio (Foxp3+ cell count divided by CD8+ cell count) as a new parameter for later analysis.

To evaluate the impact of baseline factors on TILs’ distribution, we then analyzed the correlation between parameters of TILs and clinicopathologic characteristics (Table 1). Significance was only found between MGMT status and CD8+ TILs’ density, where cases with positive MGMT status showed much more CD8+ cells’ infiltration. Although significance was not reached, higher expression of Ki-67 provided possible association with the decreased number of Foxp3+ TILs. Other correlations with differences approaching significance existed between extent of resection and CD8+ TILs, sex and Foxp3+/CD8+ ratio.

**Table 1:** Correlation of clinicopathologic characteristics with TIL parameters

	Number	Foxp3+		CD8+		Foxp3+/CD8+	
		Mean	*P*	Mean	*P*	Mean	*P*
Sex							
Male	43	7.72	0.448	47.39	0.432	0.304	0.086
Female	19	10.77		54.67		0.638	
Age (years)							
≥55	29	10.82	0.681	51.21	0.893	0.593	0.783
<55	33	6.76		48.23		0.242	
Location							
Frontal	23	10.53	0.658	58.50	0.626	0.222	0.429
Parietal	4	2.81		50.31		0.067	
Temporal	15	8.83		39.33		0.936	
Other site	7	5.59		50.95		0.195	
Combined	13	8.59		44.87		0.340	
Diameter (cm)							
≥4	31	7.72	0.740	51.12	0.725	0.205	0.745
<4	31	9.60		48.13		0.609	
Resection							
GTR	50	8.27	0.655	54.58	0.063	0.241	0.201
Non-GTR	12	10.28		28.96		1.098	
p53							
Positive	29	9.99	0.420	56.05	0.182	0.238	0.640
Negative	33	7.49		43.98		0.555	
MGMT							
Positive	22	8.54	0.525	69.53	0.009	0.215	0.971
Negative	40	8.72		38.68		0.512	
Ki-67							
Positive	23	4.76	0.058	38.06	0.374	0.204	0.106
Negative	39	10.95		56.44		0.526	

### Survival analysis based on TIL parameters

**Fig. 2:**
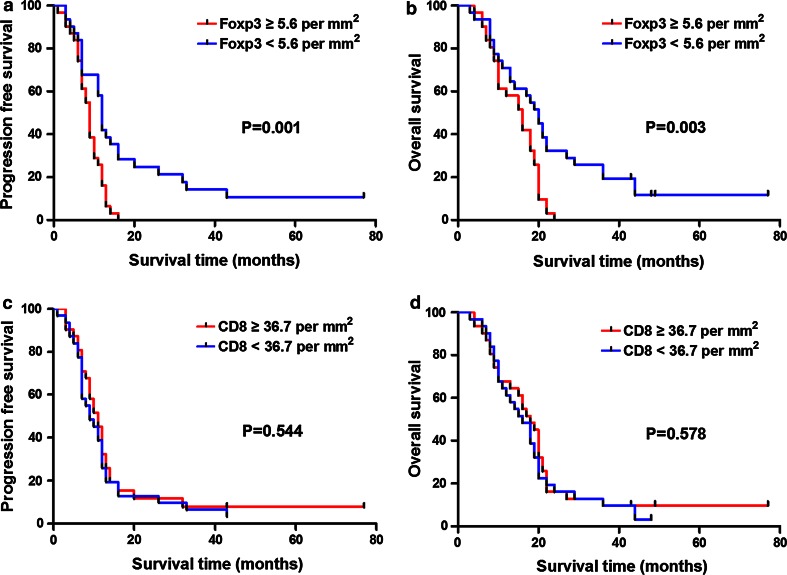
Kaplan–Meier survival curves and log-rank tests for Foxp3 and CD8 in GBM patients. **a** Higher level of Foxp3+ TIL density correlated significantly with shorter PFS. **b** Higher level of Foxp3+ TIL density correlated significantly with shorter OS. **c** PFS was not significantly different for patients with high or low level of CD8+ TIL density. **d** OS was not significantly different for patients with high or low level of CD8+ TIL density

To elucidate the prognostic effect of TIL parameters, we next dichotomized the patients into two groups using the median density as cutoff value. The median PFS was 9 months in patients with higher Foxp3 expression and 12 months in patients with lower Foxp3 expression. The difference between the two groups was significant (Fig. 2a) and OS analysis showed similar results (Fig. 2b). Comparing the two groups categorized by CD8 expression, significant difference was reached for neither PFS nor OS (Fig. 2c, d).

Considering the inherent molecular heterogeneity within GBM, we sought to analyze the prognostic significance of TIL parameters based on different molecular markers (Table 2). Higher density of Foxp3+ TILs was correlated with shortened PFS and OS regardless of MGMT status. But for Ki-67, this correlation was only valid in negative groups. CD8 expression still did not predict patient outcome in these subgroups.

**Table 2:** Prognostic significance of TIL parameters in molecular subtypes

	Foxp3		CD8	
	PFS	OS	PFS	OS
p53				
Positive	0.065	0.030	0.330	0.196
Negative	0.004	0.058	0.745	0.486
MGMT				
Positive	0.013	0.028	0.914	0.443
Negative	0.014	0.023	0.579	0.966
Ki-67				
Positive	0.649	0.570	0.597	0.414
Negative	0.000	0.021	0.402	0.254

### Prognostic significance of variables

In order to identify independent prognostic factors for patient survival, we investigated clinicopathologic characteristics as well as parameters of TILs in Cox proportional hazards regression models (Table 3). Univariate analysis was first performed and PFS was significantly different according to Foxp3+ TIL density, Foxp3+/CD8+ ratio and extent of resection. These factors together with age and tumor diameter, which gained the two least *P* values in the rest, were then adopted in model A for multivariate analysis. This model revealed that extent of resection, Foxp3+ TIL density and tumor diameter were the strongest prognostic factors. Thereafter, a second model (B) was adjusted only for the three variances and found that tumor diameter no longer showed independent prognostic value for PFS.

**Table 3:** Prognostic factors for PFS and OS in univariate and multivariate analysis

	PFS						OS					
	Univariate		Multivariate (model A)		Multivariate (model B)		Univariate		Multivariate (model A)		Multivariate (model B)	
	HR (95 % CI)	*P*	HR (95 % CI)	*P*	HR (95 % CI)	*P*	HR (95 % CI)	*P*	HR (95 % CI)	*P*	HR (95 % CI)	*P*
Sex	1.22 (0.70–2.11)	0.483					1.43 (0.82–2.48)	0.204				
Age	0.76 (0.45–1.27)	0.295	0.73 (0.43–1.26)	0.261			0.68 (0.40–1.14)	0.146	0.86 (0.49–1.51)	0.598		
Location	1.10 (0.92–1.31)	0.303					1.09 (0.91–1.29)	0.350				
Diameter	1.37 (0.81–2.31)	0.240	1.70 (0.96–3.04)	0.071	1.61 (0.92–2.83)	0.098	1.33 (0.78–2.26)	0.288				
Resection	1.96 (1.01–3.81)	0.048	2.84 (1.32–6.13)	0.008	2.54 (1.23–5.24)	0.012	2.32 (1.18–4.56)	0.015	3.09 (1.42–6.74)	0.005	2.86 (1.40–5.84)	0.004
p53	0.92 (0.55–1.55)	0.768					0.98 (0.58–1.64)	0.934				
MGMT	1.26 (0.73–2.16)	0.412					1.32 (0.77–2.28)	0.313				
Ki-67	0.79 (0.46–1.34)	0.376					0.62 (0.36–1.06)	0.078	0.59 (0.32–1.09)	0.090	0.52 (0.30–0.91)	0.021
Foxp3	0.41 (0.23–0.73)	0.002	0.33 (0.15–0.77)	0.010	0.41 (0.23–0.72)	0.002	0.45 (0.25–0.79)	0.006	0.35 (0.15–0.81)	0.015	0.42 (0.24–0.75)	0.003
CD8	1.16 (0.69–1.95)	0.570					1.15 (0.69–1.93)	0.597				
Foxp3+/CD8+	0.58 (0.35–0.99)	0.044	1.32 (0.59–2.98)	0.498			0.66 (0.39–1.11)	0.116	1.35 (0.56–3.27)	0.501		

Univariate analysis of OS indicated that Foxp3+ TIL density and extent of resection significantly coincided with patient outcome whereas no correlation was established for others. To perform multivariate analysis of OS, models were developed in the same way as described above. Foxp3+ TIL density, extent of resection and Ki-67 status were adopted in model B and all inferred as independent prognostic factors for patient OS.

## Discussion

Recently emerging articles have investigated the correlation between tumor-infiltrating Foxp3+ cells and clinical prognosis in various kinds of tumors, indicating discrepant results on how these TILs really predict patient outcome. To settle this controversy, DeLeeuw et al. [10] made great efforts in meta-analyzing the prognostic significance of Foxp3+ TILs across 16 non-lymphoid cancers by enrolling 58 studies. Assessing several technical and biologic factors, they finally proved two causes, tumor site and use of multiple markers, responsible for these debatable claims. Accordingly, Foxp3+ TILs coincided with generally favorable prognosis in colorectal cancer but poor prognosis in hepatocellular cancer. However, studies concerning glioma or even GBM were really rare and not included in the systematic review.

In 2008, Heimberger et al. [11] for the first time analyzed prognostic impact of Foxp3+ TILs in human glioma based on immunohistochemical staining and reached no statistical significance between 25 patients with Foxp3 presence and 27 ones with Foxp3 absence. This conclusion was later confirmed by Jacobs et al. [12] using flow cytometry. Nevertheless, the second study only enrolled 29 GBM patients and the comparison performed by log-rank test showed marginal significance (*P* = 0.06). Alternatively, Wiencke et al. [21] revealed that patients with low Foxp3 expression gained a significantly prolonged survival by quantitative methylation specific PCR, and it was lately confirmed by Wang et al. [13] on immunohistochemistry. Moreover, Fong et al. [22] from U.S. suggested that in patients who received DC vaccination, decreased frequencies of Tregs after the treatment predicted extended survival.

Our study in this article had the largest population to date and suggested that Foxp3 might be an independent adverse prognostic factor. The controversy among these studies may be explained as follows. First, various methods to identify Tregs, including flowcytometry and immunohistochemistry, may lead to different results. Generally, flowcytometry was more accurate in determining whether the Foxp3+ cells were really Tregs by using multiple staining. However, staining strategy also varied between studies performing flowcytometry that Heimberger et al. defined CD4+ CD25+ Foxp3^high^CD127^low^ cells as Tregs while Fong et al. detected CD3+ CD4+ CD25+ CD127^low^ ones. Second, cutoff values were different among these studies. For example, Heimberger, et al. used the presence/absence of Foxp3+ TILs to determine the clinical significance, but our study dichotomized patients by the median of Foxp3 density. More complicated methods such as recursive partitioning survival tree were performed by Fong et al. Therefore, to make a more convincible comparison between different studies, an accurate and reasonable cutoff value should be developed and widely accepted in the future. Third, the populations enrolled in these studies were obviously heterogeneous. Subtypes of GBM, anti-tumor treatment or even race, which usually influenced patient outcome, were not unified, thus increasing inter-study bias. In addition, other technical details should never be ignored, encompassing the antibodies used, evaluation strategy and statistical analysis.

CD8+ TILs, also known as cytotoxic T lymphocytes (CTL), have long been believed to take a major part in adaptive immunity against tumor invasion, thus theoretically prolonging patient survival and having great potential as a prognostic indicator. In support, both Yang et al. [23] and Kim et al. [24] found a better outcome in GBM patients with higher CD8+ cells infiltration. Intriguingly, many studies contradicted the hypothesis and showed no relationship between CD8+ TILs and clinical outcome [25, 26]. It was even reported by Wiencke et al. [21] that more CD3+ TILs in GBM tissues, most of which were CD8+, predicted poorer prognosis. In accordance, there remained no significant difference in our study when comparing PFS or OS according to CD8 dichotomization. These findings together indicated that different activation modes of CD8+ TILs might coexist and were not distinguished by immunohistochemical staining. Furthermore, as a positive correlation was discovered between Foxp3 and CD8 expression in some studies [17, 27], CD8+ TILs’ interactions with inhibitory T cells as well as other immune participators such as microglias/macrophages should be taken into account. In support, it was reported that Tregs could compromise CTLs’ function via immunosuppressive cytokines such as IL-4, TGF-β or direct cell to cell contact [28]. Our results that the density of Foxp3 instead of CD8 was perhaps significant for survival may imply that suppressive capacity of Tregs was sufficient to override the anti-tumor immune response initiated by CD8+ TILs in GBM microenvironment. So further investigation of the crosstalk between Tregs and CD8+ TILs will pave the way for developing effective immunotherapy to inhibit glioma propagation.

Integrating both subtypes and probably reflecting more comprehensive immune pattern, the ratios such as Foxp3+/CD8+ were frequently chosen as candidates for independent prognostic factors and led to positive findings in several cancers. It was reported that the ratio of regulatory and cytotoxic T cells rather than their absolute numbers had greater impact on tumor growth in hepatocellular carcinoma [29]. Higher CD8+/Foxp3+ was authenticated to be associated with favorable outcome in both breast cancer and ovarian cancer [25, 30]. Partially supporting these results, we also found that higher Foxp3+/CD8+ was likely to predict shorter PFS in GBM patients but when adjusted by other factors, it was no longer significant. Moreover, neither univariate nor multivariate analysis suggested the ratio as prognosis factor for OS. The positive correlation between expression of Foxp3 and CD8 might compromise their ratio’s prognostic value compared with single Foxp3. Still and all, the ratio of subtypes should be included in future research allowing for its potential effect.

Instead of a single entity, cancer is a heterogenous disease with different profiles of genetic and epigenetic alterations, which may influence immune cells’ behavior in interdisciplinary microenvironment and thus prognostication [31]. In support, Frey et al. [32] identified frequency of Foxp3+ TILs as an independent prognostic factor in mismatch repair-proficient colorectal cancer other than mismatch repair-deficient colorectal cancer. Additionally, intratumoral Tregs were demonstrated to predict worse OS in cyclooxygenase-2-positive uveal melanoma while the relationship between Foxp3+ cells and clinical outcome has been tested repeatedly in breast cancer subtypes according to estrogen receptor [25, 33, 34]. Our research was the first to explore Foxp3+ TILs’ prognostication based on molecular classifications in GBM and selected common biomarkers such as p53, MGMT and Ki-67 for analysis. Of note, we found that Foxp3 could show stronger prognostic significance in Ki-67− group compared with Ki-67+ group, and elucidation of the underlying mechanisms relies on further experiments targeting at cross-talk among these molecular.

There are some limitations of this study. First, although bigger than previous ones, our study sample was still limited to obtain enough differences between groups, which might partially explain that only Foxp3 density and extent of resection were identified as potential prognostic factors for PFS and OS. Second, since it has been recently reported that glioma cells also expressed Foxp3, our data based on immunohistochemistry were prone to interpreting the biologic behavior of Foxp3 expression more than exact TILs. For this, double or multiple staining plus specific markers such as CD4 and CD25 will be needed in later investigation. Third, we only detected Foxp3+ and CD8+ cells but as there existed complicated interactions among various immune cells in tumor microenvironment, it would be more reasonable to include all TIL subsets in multivariate models.

In conclusion, the present study observes poorer survival in GBM patients with more Foxp3+ TILs, and indicates the density of Foxp3+ TILs as a possible prognostic factor via multivariate models. Furthermore, our results suggest that evaluation of Foxp3+ TILs in combination with various molecular classifications might improve the prognostic stratification of GBM. Allowing for several limitations, larger studies are still needed in the future to provide stronger evidence for utilizing Foxp3 as a candidate biomarker or even implementing Foxp3+ TILs as novel target of immunotherapy.

## Electronic supplementary material

Below is the link to the electronic supplementary material.

Supplementary material 1 (EPS 8958 kb)
